# Streamlining Recruitment for AD Clinical Trials: Concurrent Detection of Cognitive Impairment and Amyloid‐Beta PET Status with a Machine Learning‐Enabled Digital Cognitive Assessment

**DOI:** 10.1002/alz70861_108911

**Published:** 2025-12-23

**Authors:** Ali Jannati, Karl Thompson, Claudio Toro‐Serey, Connor Higgins, John Showalter, David Bates, Sean Tobyne, Alvaro Pascual‐Leone

**Affiliations:** ^1^ Linus Health, Boston, MA USA; ^2^ Hebrew SeniorLife, Boston, MA USA; ^3^ Harvard Medical School, Boston, MA USA

## Abstract

**Background:**

Identifying suitable participants for clinical trials of disease‐modifying treatments (DMTs) for Alzheimer’s disease (AD) is impeded by high screening failure rates and costly prescreening. Such identification requires both early detection of cognitive impairment and abnormal brain amyloid‐beta (Aβ) status. Therefore, an efficient and cost‐effective solution to streamline the process of early AD identification can significantly streamline the process of participant recruitment for clinical trials of DMTs for AD. In this study, we assessed the accuracy of a brief digital cognitive assessment, the Linus Health Digital Clock and Recall (DCR), to identify cognitive impairment and predict brain amyloid‐beta (Aβ) status on positron emission tomography (PET).

**Method:**

930 participants (mean age 72.0±6.7; 56.8% female; 23% minorities) in the Bio‐Hermes‐001 study were classified as cognitively unimpaired, mild cognitive impairment, or probable Alzheimer’s dementia, and 35.1% were Aβ+ on 18F‐florbetapir PET scan. A DCR‐based algorithm (*LinusAD*) used age, APOE status, drawing metrics, speech and acoustic features, and temporal‐spatial features of stylus manipulation. LinusAD was compared with MMSE and blood‐based biomarkers (BBMs) Lilly pTau‐217, Quanterix pTau‐181, C2N Aβ42/40, and C2N Amyloid Probability Score (APS). Non‐inferiority was established if a 95% confidence interval of the bootstrapped AUC difference between two tests was within ΔAUC±0.1.

**Result:**

Cognitive‐impairment identification by the DCR (AUC=0.85) was superior to Aβ42/40, pTau‐181, and pTau‐217 (AUCs=0.63, 0.66, 0.72) and non‐inferior to RAVLT and MMSE (AUCs=0.89, 0.82). Aβ‐status prediction by LinusAD (AUC=0.882) was equivalent to BBMs (AUC=0.775–0.887; average AUC=0.82) and superior to MMSE (AUC=0.71). Combining the DCR with Aβ42/40, pTau‐181, and pTau‐217 improved their Aβ‐status prediction performance to AUCs of 0.882, 0.835, and 0.905, respectively.

**Conclusion:**

The 3‐minute DCR was superior to BBMs in detecting cognitive impairment and boosted their Aβ‐PET prediction ability to levels comparable to CSF biomarkers of AD. By leveraging AI process metrics, the DCR enables the cost‐effective identification of the most suitable participants for novel DMTs and can decrease high screen‐failure rates in AD clinical trials.

